# c-Myc Protein Level Affected by Unsymmetrical Bisacridines Influences Apoptosis and Senescence Induced in HCT116 Colorectal and H460 Lung Cancer Cells

**DOI:** 10.3390/ijms23063061

**Published:** 2022-03-11

**Authors:** Monika Pawłowska, Jolanta Kulesza, Ewa Augustin

**Affiliations:** Department of Pharmaceutical Technology and Biochemistry, Faculty of Chemistry, Gdańsk University of Technology, 80-233 Gdańsk, Poland; monpawlo@pg.edu.pl (M.P.); jolanta.kulesza@pg.edu.pl (J.K.)

**Keywords:** unsymmetrical bisacridines (UAs), G-quadruplexes, c-Myc, apoptosis, senescence, drug discovery, anticancer potential, biological activity

## Abstract

Unsymmetrical bisacridines (UAs) are highly active antitumor compounds. They contain in their structure the drugs previously synthesized in our Department: C-1311 and C-1748. UAs exhibit different properties than their monomer components. They do not intercalate to dsDNA but stabilize the G-quadruplex structures, particularly those of the *MYC* and *KRAS* genes. Since *MYC* and *KRAS* are often mutated and constitutively expressed in cancer cells, they can be used as therapeutic targets. Herein, we investigate whether UAs can affect the expression and protein level of c-Myc and K-Ras in HCT116 and H460 cancer cells, and if so, what are the consequences for the UAs-induced cellular response. UAs did not affect K-Ras, but they strongly influenced the expression and translation of the c-Myc protein, and in H460 cells, they caused its full inhibition. UAs treatment resulted in apoptosis, as confirmed by the morphological changes, the presence of sub-G1 population and active caspase-3, cleaved PARP, annexin-V/PI staining and a decrease in mitochondrial potential. Importantly, apoptosis was induced earlier and to a greater extent in H460 compared to HCT116 cells. Moreover, accelerated senescence occurred only in H460 cells. In conclusion, the strong inhibition of c-Myc by UAs in H460 cells may participate in the final cellular response (apoptosis, senescence).

## 1. Introduction

**Figure 1:**
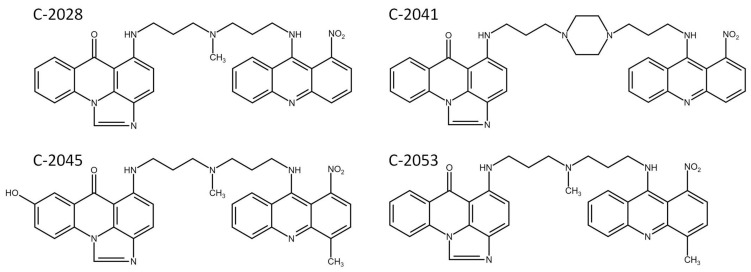
The chemical structures of UA derivatives: C-2028, C-2041, C-2045, and C-2053.

Unsymmetrical bisacridines (UAs) are a group of new, promising antitumor compounds among acridine derivatives, developed at Gdańsk University of Technology. They exhibit high cytotoxic activity against a lot of tumor cells in vitro, as well as high antitumor efficacy against several types of human cancer xenografts in nude mice, including lung and colorectal cancers [1,2,3]. UAs, whose structures are presented in Figure 1, contain elements of monomeric compounds previously synthesized in our Department: imidazoacridinone, C-1311 and 1-nitroacridine, C-1748, connected by diverse linkers.

Our previous studies have shown that UAs exhibit different properties than their monomeric components. They do not intercalate to dsDNA, but they interact with quadruplex DNA [3]. G-quadruplexes (G4) are present in specific human guanine-rich sequences with functional significance, such as telomeres, oncogene-promoter regions, including *MYC*, *RAS*, *KIT*, *BCL2*, *VEGF* genes and 5′- and 3′-untranslated region (UTR) of mRNA [4]. They have been shown to be a regulatory motif in a number of critical cellular processes, such as gene transcription, translation, replication and genomic stability. 

*MYC* plays a key role in growth control, differentiation and apoptosis, therefore its abnormal expression has been associated with many tumors [5,6,7]. Since *MYC* gene is often mutated and constitutively expressed in cancer cells, it can be used as a therapeutic target [8,9]. c-Myc is expressed in cycling cells, and only transient inhibition can lead to tumor regression. Its overexpression or inhibition can sensitize cells to traditional chemotherapeutic agents, leading to induction of apoptosis [10]. The decision of a cell to undergo apoptosis and the way *MYC* regulates this apoptotic response depends on the specific cell type and the physiological status of the cell [11]. It is also well known that suppression of the *MYC* oncogene induces cellular senescence in diverse tumor types, including lymphoma, osteosarcoma and hepatocellular carcinoma [12]. The expression of *MYC* is tightly controlled in normal cells by a range of upstream and downstream mechanisms at the genetic, mRNA and protein levels, but it becomes dysregulated and overexpressed in over 70% of human cancers [13]. This may be driven by a number of mechanisms at the DNA, RNA and protein levels, although rarely by direct *MYC* mutation [14]. In the case of the *MYC* gene, G-quadruplexes regulate gene expression, including 90% of *MYC* expression [15], thus making it a potential drug target for *MYC*-deregulated cancer. Small molecules that stabilize G-quadruplexes formed in the *MYC* promoter have been shown to inhibit their expression, indicating a potential therapeutic possibility of targeting G-quadruplex promoters to modulate transcription [16]. For example, quindolines [17] and actinomycin D [18] have been reported to suppress *MYC* transcription by stabilizing the *MYC* G-quadruplex promoter.

The *RAS* gene (*KRAS*, *NRAS* and *HRAS*) represents the most frequently mutated oncogenes in human cancers, in particular in pancreatic, lung and colorectal cancers [19]. It plays an important role in cell proliferation, differentiation and survival. However, *RAS* remains one of the less successful therapeutics targets, with limited treatments available and considerable side effects [20]. Therefore, new therapies focus not only directly on the K-Ras protein but also on the *RAS* gene and its promoter regions, the G-quadruplexes [21]. 

Considering the above, the aim of this study was to evaluate whether unsymmetrical bisacridines could affect the expression and protein level of c-Myc and K-Ras in human colon HCT116 and lung H460 cancer cells. These cell lines were selected due to their high sensitivity to the tested unsymmetrical bisacridines [3]. In addition, we wanted to investigate whether different levels of c-Myc and K-Ras proteins have an impact on final UAs-induced cellular response (apoptosis and senescence) in these cancer cells. 

## 2. Results

### 2.1. Cytotoxic Effects of UA Compounds against Colon and Lung Cancer and Normal Cells

The cytotoxicity of new unsymmetrical bisacridines was evaluated in four cell lines: two cancer, colorectal HCT116 and lung H460, and two normal colon epithelial CCD 841 CoN and lung fibroblast MRC-5. Cells were treated with the four bisacridine derivatives for 72 h at concentrations ranging from 0.00001 to 10 µM, which resulted in concentration-dependent inhibition of cell proliferation. The obtained IC_50_ and IC_90_ values are presented in Table 1. 

All four bisacridine compounds exhibited very high cytotoxicity against the two cancer cell lines, and the sensitivity of HCT116 and H460 cells was very similar. C-2028 and C-2041 derivatives inhibited the proliferation of both cancer cell lines at very low concentrations; IC_50_ and IC_90_ values did not exceed 0.016 and 0.05 µM, respectively. Considering IC_90_ values, C-2045 was about 10 times less active than the previous two compounds, while C-2053—5 times.

Normal cells, CCD 841 CoN and MRC-5, were less sensitive to UAs than cancer cells. The calculated IC_50_ values for the four compounds ranged from about 0.02 µM for C-2028 to 0.3 µM for C-2045 and were quite similar for both cell lines. Furthermore, IC_90_ values determined for four UAs against normal cells exceeded 2.7 µM and were approximately 100 times higher for C-2028 and C-2041 compounds, and 10 times for C-2045 and C-2053 than against cancer cell lines.

### 2.2. c-Myc and K-Ras Expression in Cancer and Normal Cells

In our previous work [3] we showed that unsymmetrical bisacridines are potent G-quadruplex stabilizers. G-quadruplexes, G4, have been found in telomeric DNA [22], as well as in the promoter regions of a number of genes, including cancer oncogenes, such as *MYC* [23] and *KRAS* [24]. G4 are also present in regions of RNA [25]. Our earlier results indicated that UA compounds strongly stabilized G-quadruplex structures, particularly those of the *MYC* and *KRAS* genes. Therefore, we decided to check the influence of our bisacridine compounds on the expression of *MYC* and *KRAS* genes in HCT116 and H460 cells. The examination was performed using real-time polymerase chain reaction analysis (RT-PCR). 

**Figure 2:**
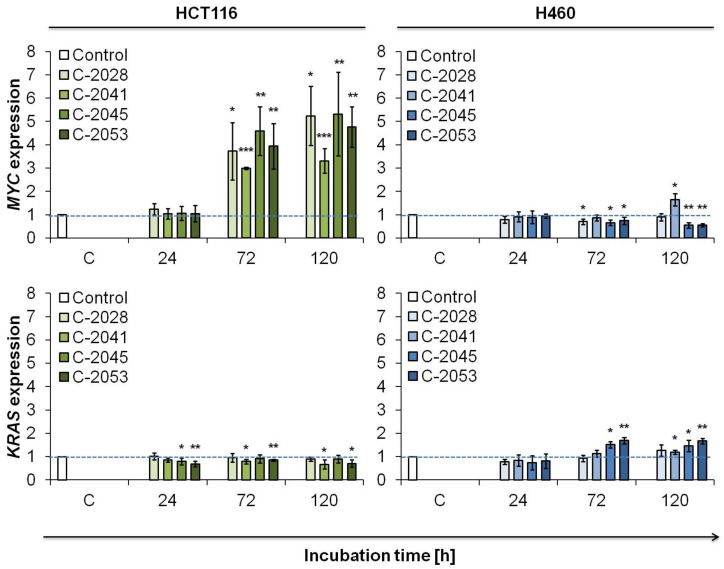
*MYC* and *KRAS* expression in HCT116 and H460 cells. Cells were incubated with IC_90_ doses of UAs for the times indicated. Total mRNA was isolated, transcribed to cDNA, and real-time PCR analysis was performed with the appropriate primers for *MYC* and *KRAS* genes. *ACTB* (β-actin) was used as housekeeping gene standard. Relative gene expression was calculated using 2^−ΔΔCt^ method [26]. Significantly different from the control at: * *p* < 0.05; ** *p* < 0.01; *** *p* < 0.001; (*n* ≥ 3).

The obtained results showed that in HCT116 colon cancer cells treated with UA compounds, the *MYC* expression strongly increased after 72 h of incubation and remained at a similar level after 120 h (Figure 2). The upregulation was the strongest in C-2045-exposed cells (5.3-fold increase after 120 h) and the weakest in cells treated with C-2041 (3.3-fold increase). *KRAS* expression in HCT116 cells incubated with UAs decreased slightly compared to untreated control cells; however, a significant difference was observed only in the case of C-2053 derivative. UA compounds showed weak impact on the transcription of *KRAS*.

In H460 lung cancer cells, the evaluation of UA compounds’ influence on *MYC* and *KRAS* gene expression gave different results than in colon cells (Figure 2). The mRNA level of *MYC* gene decreased just after 24 h of incubation with all tested bisacridine derivatives to the level of about 0.9. The expression of *MYC* gene in H460 cells exposed to C-2028 stayed at the similar level with the time of incubation. However, in C-2041 treated cells, *MYC* expression increased strongly to 1.65 after 120 h. In contrast, mRNA level of *MYC* gene in H460 cells incubated with C-2045 and C-2053 decreased to around 0.55. Furthermore, in H460 cells, *KRAS* expression remained almost at the same level through the whole time of incubation with C-2028 and C-2041 compounds. Importantly, in C-2045 and C-2053 H460 treated cells, the relative amount of *KRAS* mRNA increased significantly to around 1.5 and 1.7, respectively. 

The expression of *MYC* and *KRAS* was also checked in normal cells, CCD 841 CoN and MRC-5, treated with four bisacridines for 120 h. The level of *MYC* and *KRAS* mRNA in colon CCD 841 CoN cell was slightly elevated, and this increase ranged between 1.3 to 1.8 for the particular gene or compound treatment. In lung MRC-5 cells, bisacridine derivatives did not affect the expression of both *MYC* and *KRAS* genes (Figure S1 in the Supplementary Materials). 

### 2.3. Western Blotting Analysis of c-Myc and K-Ras Level in Cancer and Normal Cells

**Figure 3:**
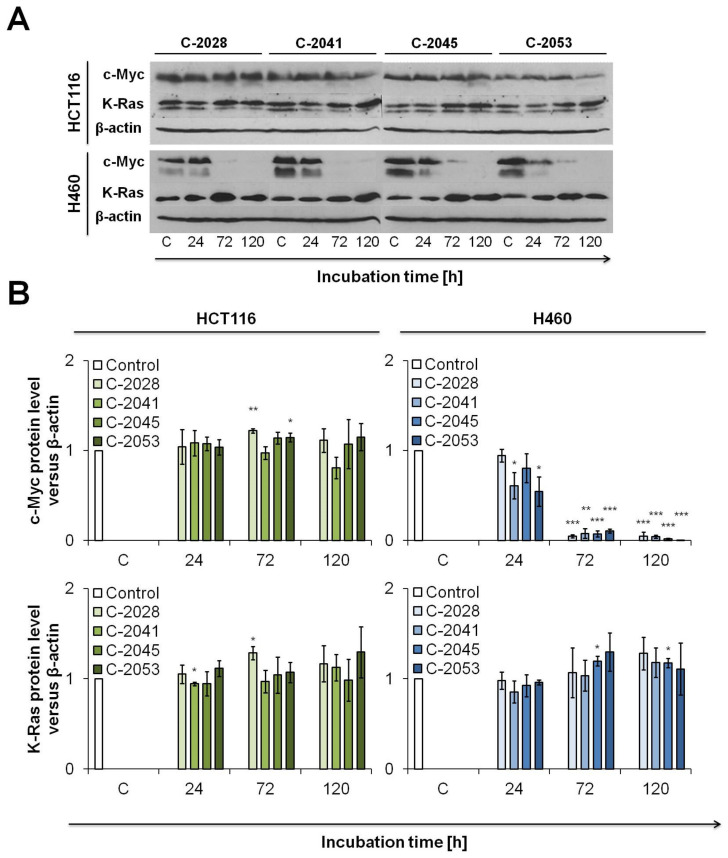
Western blot analysis of c-Myc and K-Ras protein levels in HCT116 and H460 cells. Cells were incubated with IC_90_ doses of UAs for the times indicated. Whole cell extracts were prepared, 20 μg of proteins/lane were separated by polyacrylamide gel electrophoresis and semi-dry transferred on a membrane. Protein levels were detected after immunostaining the membrane with appropriate antibodies and ECL developing. (**A**) Representative Western blot analysis of c-Myc and K-Ras in HCT116 and H460 cells and (**B**) their relative densitometry quantification performed using ImageJ Software. Significantly different from the control at: * *p* < 0.05; ** *p* < 0.01; *** *p* < 0.001; (*n* ≥ 3).

The interaction of bisacridines with c-Myc and K-Ras was also investigated at the protein level, since G4 can also be observed in RNA regions and may influence the translation process [25]. The results obtained from the Western blot analysis are shown in Figure 3. In HCT116 colon cells, the level of c-Myc protein did not change during the time of incubation with all UA compounds. However, the c-Myc protein level completely decreased in H460 lung cells from 72 h of incubation with all UAs (Figure 3). These results are opposite to those obtained from RT-PCR analysis, where in HCT116 cells, with time of incubation with UAs, the level of *MYC* mRNA strongly increased, whereas the level of protein did not change. Concomitantly, in H460 cells, the exposure to UAs did not cause considerable alterations in the mRNA level of c-Myc, but the protein level significantly decreased. Summarizing the above, UA derivatives may have a strong impact on the process occurring between transcription and translation of the c-Myc protein. 

In both HCT116 and H460 cell lines, the level of K-Ras protein increased slightly with the time of incubation with UAs, especially in cells treated with C-2045 and C-2053 (Figure 3). These results are consistent with RT-PCR analysis in which, in particular in H460 cells, the mRNA level was also slightly elevated (Figure 2). Furthermore, in normal cells, CCD 841 CoN and MRC-5, the bisacridine compounds did not alter the c-Myc and K-Ras protein levels after 120 h of treatment (Figure S2 in the Supplementary Materials).

### 2.4. Cell Cycle Distribution of Colon and Lung Cancer and Normal Cells Treated with UAs

In the next stage of our study, we wanted to determine the cellular mechanism of action of our bisacridine derivatives in colon and lung cells and establish the relationship between the induced response and the level of c-Myc protein. Firstly, we analyzed the influence of UAs on cell cycle progression, and then we focused on the study of cell death induction in cancer and normal cells.

**Figure 4:**
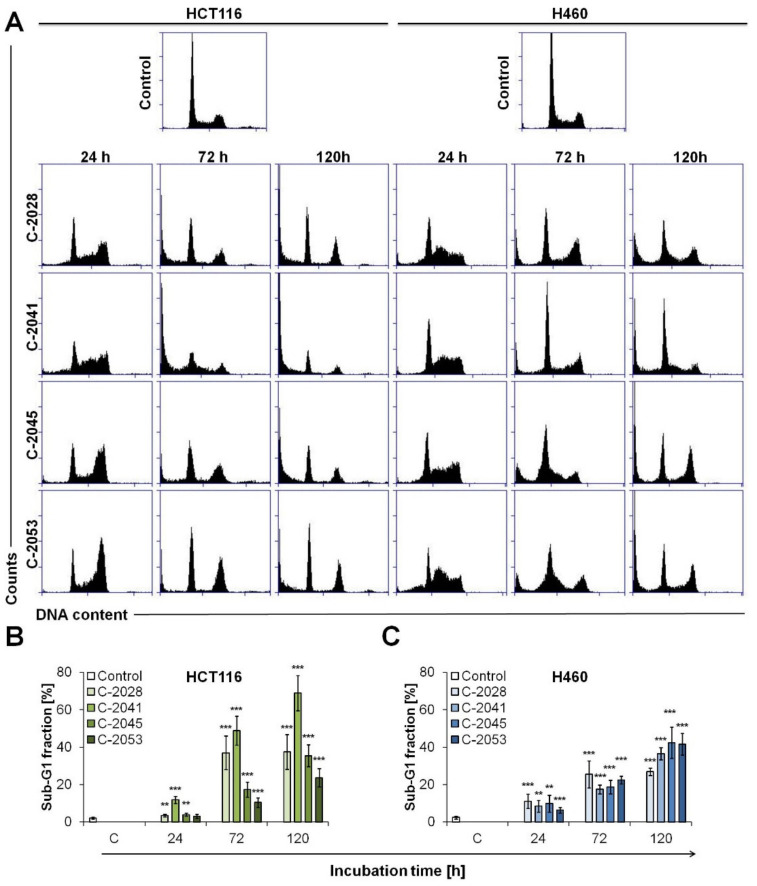
Cell cycle distribution of cancer, HCT116 and H460 cells. Cells were untreated (control) or treated with 0.04, 0.05, 0.4 and 0.2 µM of C-2028, C-2041, C-2045 and C-2053 compounds, respectively, for the times indicated and subjected to propidium iodide staining and flow cytometry analysis, as described in Materials and Methods. (**A**) Histograms show the number of cells (*y*-axis) versus DNA content (*x*-axis) and are representative of at least three experiments for each condition. Bar graphs show quantified data, expressed as the percentages of HCT116 (**B**) and H460 (**C**) cells with less than 2N DNA (sub-G1 fraction). Significantly different from the control at: * *p* < 0.05; ** *p* < 0.01; *** *p* < 0.001; (*n* ≥ 4).

HCT116 and H460 cancer cells were treated with UAs for 24, 72 and 120 h, while normal cells, CCD 841 CoN and MRC-5, for 120 h at concentrations equal to 0.04, 0.05, 0.4 and 0.2 µM for C-2028, C-2041, C-2045 and C-2053, respectively. Cell cycle distribution of cancer cells exposed to UAs is presented in Figure 4. Treatment with bisacridine derivatives led in HCT116 cells to a decrease in the content of cells in G1 phase from 48.5% in untreated control cells to about 22–29% after 24 h of exposure to UAs (Table S1 in the Supplementary Materials). G1 phase content of HCT116 cells did not differ much during prolonged time of incubation with drugs, with the exception of C-2041, which led to a reduction in 2N DNA content, from 29 to 13.6%. After 24 h of treatment with UAs, the content of cells in the S phase stayed at quite similar level, increasing slightly, especially for C-2041, from 16.7 to 26%. After 72 h of incubation, the number of dividing cells dropped to less than 5% after 120 h of treatment with all UAs. Three compounds, C-2028, C-2045 and C-2053, induced a slight accumulation of cells in G2/M phase in HCT116 cells after 24 h of exposure, and during the treatment, the content of 4N DNA in cells dropped to around the initial percentage (23%). In turn, the sub-G1 fraction increased during the time of incubation, and after 120 h it reached 37.5, 68.9, 35.4 and 23.6% for C-2028, C-2041, C-2045 and C-2053, respectively (Figure 4B). C-2041 led to the degradation of DNA to the greatest extent. Furthermore, the content of polyploid cells differed during the time of incubation of HCT116 cells with UAs compared to control cells. C-2028 and C-2041 caused a decrease in the number of cells with more than 4N DNA (from 10 to 5%), while C-2045 and C-2053 increased this fraction from 10 to around 15%. 

The distribution of H460 cells in the cell cycle presented many differences between the control and UAs treated cells (Figure 4). Bisacridine derivatives caused a decrease in cell content in the G1 phase, which dropped from 60% to approximately 30–40% after 24–72 h of treatment. C-2045 and C-2053 induced a more profound reduction in the content of cells with 2N DNA, and after 120 h, the fraction of cells in the G1 phase was around 25%. All UAs derivatives induced conspicuous accumulation of cells in the S phase after 24 h of exposure in H460 cells. The number of cells with 2–4N DNA increased from 16.5 in control cells to about 30% in treated cells, and the greatest increase was observed in lung cancer cells incubated with C-2041 (34.8%, Table S1 in the Supplementary Materials). The prolonged bisacridines treatment led to a reduction in the number of cells in the S phase, and after 120 h, it reached around 10% of H460 population. All UAs caused very slight accumulation of H460 cells in the G2/M phase of cell cycle. Only in C-2041 treated cells, the population of cells with 4N DNA dropped below the level observed in the control cells. Most importantly, all four UA compounds induced DNA degradation in H460 cells. During the time of incubation, the sub-G1 fraction increased, and after 120 h, it reached 26.8, 36.5, 42.4 and 41.5% in cells treated with C-2028, C-2041, C-2045 and C-2053, respectively (Figure 4C). It is worth to mention that UAs did not cause any change in the content of H460 cells with more than 4N DNA, and the population of polyploid cells remained very low (around 2%) throughout incubation.

**Figure 5:**
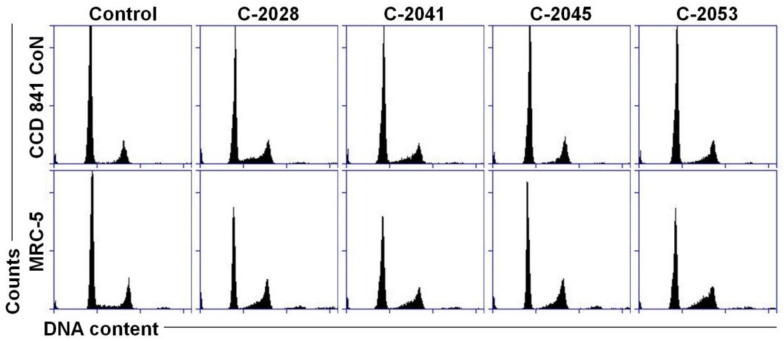
Cell cycle distribution of normal CCD 841 CoN and MRC-5 cells. Cells were untreated (control) or treated with 0.04, 0.05, 0.4 and 0.2 µM of C-2028, C-2041, C-2045 and C-2053 compounds, respectively, for 120 h, and subjected to propidium iodide staining and flow cytometry analysis, as described in Materials and Methods. Histograms show the number of cells (*y*-axis) versus DNA content (*x*-axis) and are representative of three experiments for each condition (*n* = 3).

Unlike in cancer cells, UAs after 120 h of treatment did not cause much alteration in the distribution of normal cells in the cell cycle (Figure 5). The histograms obtained for treated CCD 841 CoN and MRC-5 cells looked quite similar to those for the controls. Only the sub-G1 fraction increased slightly after bisacridines exposure, from around 2% in control cells to 6% in treated cells. 

### 2.5. Morphological Changes of Nuclei Triggered by UAs in Cancer and Normal Cells

**Figure 6:**
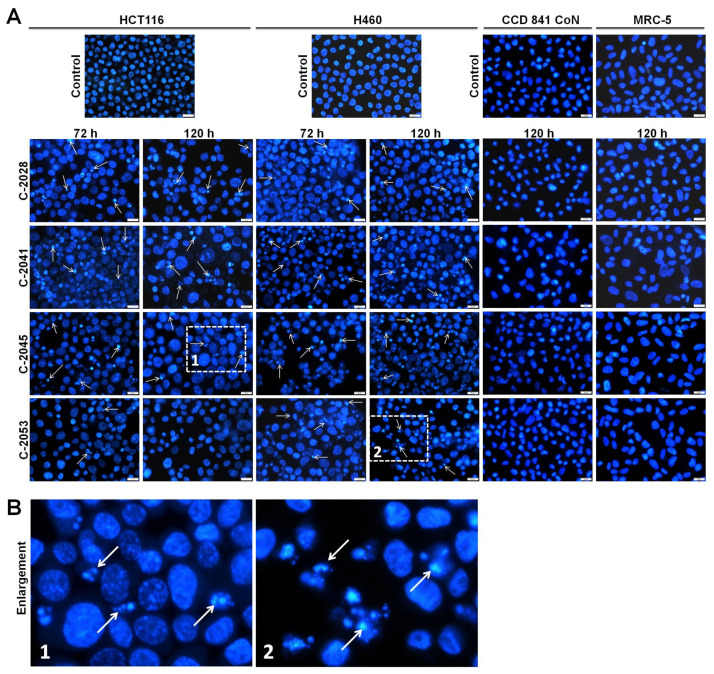

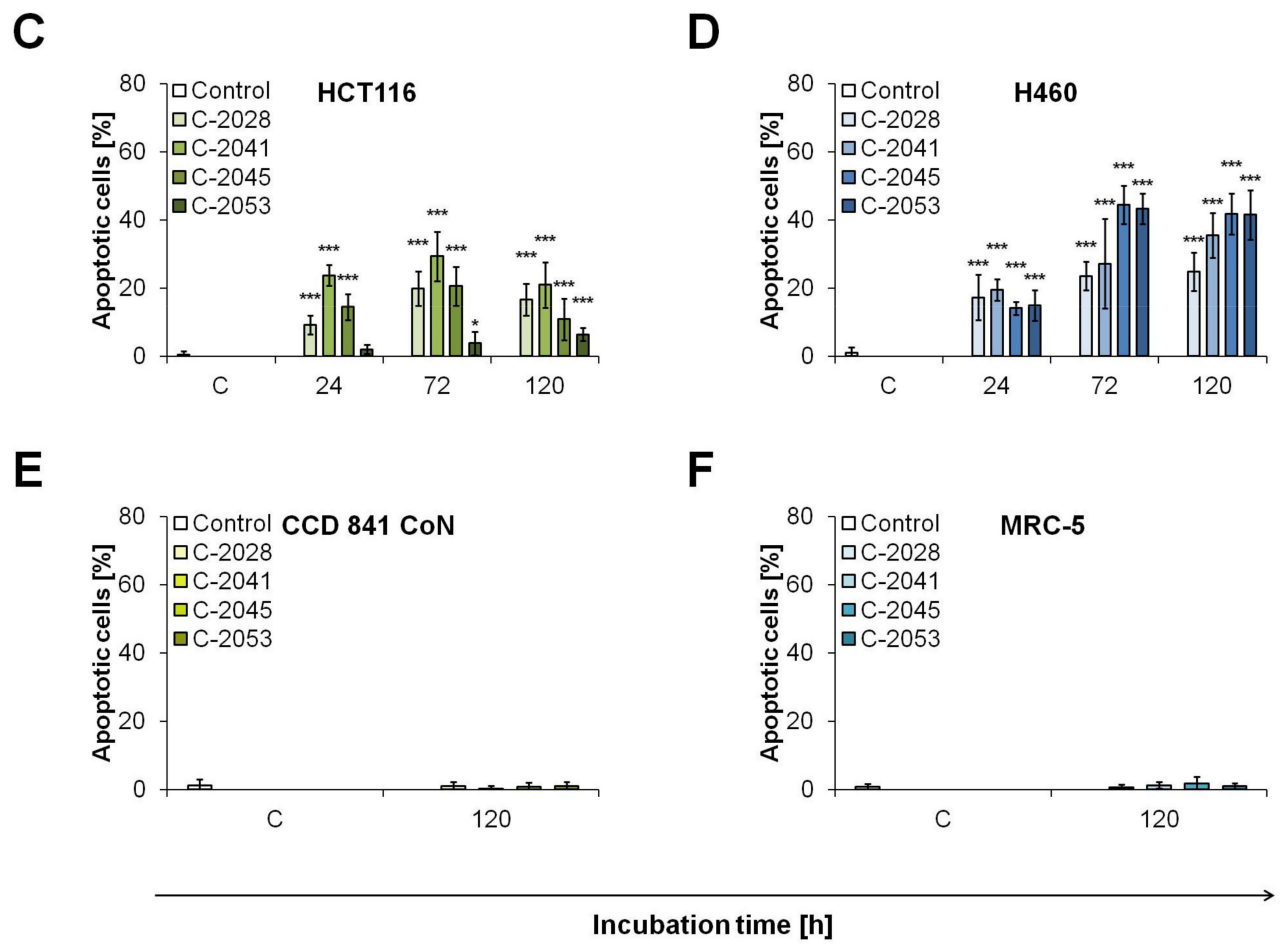
Morphological changes of nuclei of cancer and normal cells treated with UAs. (**A**) Pictures (representative of three independent experiments) present changes in nuclear morphology of HCT116, H460, CCD 841 CoN and MRC-5 cells treated with 0.04, 0.05, 0.4 and 0.2 µM of C-2028, C-2041, C-2045 and C-2053 compounds. Cells were stained with Hoechst 33342 (1 mg/mL) and visualized under fluorescent microscope (400× magnification). Arrows point at the cells with changes in the nuclei characteristic of apoptosis (condensed, intensely stained, fragmented chromatin). White scale bars presented on images correspond to 20 µm (**B**) Enlarged fragments of pictures with indicated changes characteristic of apoptosis. (**C**–**F**) Bar graphs showing quantified data, expressed as the percentages of apoptotic cells established for HCT116 (**C**), H460 (**D**), CCD 841 CoN (**E**), and MRC-5 (**F**) cells. Significantly different from the control at: * *p* < 0.05; ** *p* < 0.01; *** *p* < 0.001; (*n* ≥ 3).

To determine the cellular response induced by UAs in cancer and normal cells, morphological observation of nuclei was conducted. Cells were treated with 0.04, 0.05, 0.4 and 0.2 µM of C-2028, C-2041, C-2045 and C-2053 compounds, respectively, and after fixation, cells were stained with Hoechst 33342 and visualized under a fluorescent microscope (Figure 6A). As the incubation time increased, HCT116 cells treated with UAs became larger, especially those after 120 h and exposed to C-2041 and C-2045 compounds. The most profound change in the nuclei morphology was the appearance of cells with condensed chromatin, forming one nucleus, the so-called pycnotic cell, or fragmented into discrete bodies. In some cells, condensing chromatin was located in the nuclear periphery. These alterations in HCT116 cells treated with UAs suggested the induction of apoptosis and the formation of apoptotic bodies (Figure 6B). The highest number of apoptotic cells was observed in cells treated with C-2041 and C-2045, especially after 72 h (29.4 and 20.6% for C-2041 and C-2045, respectively; Figure 6C). In cells exposed to C-2053, it was difficult to find cells undergoing apoptosis; only a few appeared after 72 and 120 h treatment, up to 6.4%. Moreover, the increase in the number of polyploid cells during the cell cycle analysis of HCT116 cells exposed to C-2045 and C-2053 might have suggested the induction of mitotic catastrophe. Few multinucleated cells (a hallmark of mitotic catastrophe) could be found in the case of C-2045-HCT116 treated cells. 

Microscopic observations revealed that the nuclei of H460 cells exposed to UAs underwent many alterations (Figure 6A). Many stained nuclei exhibited changes typical of apoptosis, such as chromatin condensation and fragmentation into apoptotic bodies (Figure 6B). The alterations appeared at a similar level for cells incubated for 72 and 120 h. Most profound induction of apoptosis was observed in H460 cells treated with C-2045 (41.8%) and C-2053 (41.6%), while treatment of cells with C-2028 caused the least degradation (only 24.8% apoptotic cells). Additionally, the exposure of H460 cells to C-2028 and C-2053 led to an enlargement of the nuclei in comparison to the control and C-2041 and C-2045 treated cells. The effect was observed mainly after 72 h of incubation. 

The nuclei morphology of normal CCD 841 CoN and MRC-5 cells did not change significantly after prolonged incubation with UA compounds (Figure 6A). After 120 h of exposure to UAs, almost no features characteristic of apoptosis were observed (only 1–2% of cells looked apoptotic; Figure 6E,F). Only C-2041 treated cells of both cell lines became enlarged. 

### 2.6. UAs Induction of Apoptosis in Cancer Cells

Cell cycle analysis revealed that in HCT116 and H460 cells, UAs treatment led to degradation of DNA, which may indicate the induction of apoptosis (Figure 4). Additionally, nuclei observation showed that, in HCT116 and H460 cells, characteristic apoptotic changes also occurred (condensed and fragmented chromatin, Figure 6). Therefore, in the next step, several tests were carried out to evaluate the cellular response, in particular the induction of apoptosis triggered by UA compounds in colon and lung cancer cells. Externalization of phosphatidylserine from the inner to outer leaflet of the cell membrane (annexin V–FITC staining), disruption of cell membrane (PI staining), caspase-3 activation and subsequent PARP cleavage (flow cytometry immunostaining) and decrease in mitochondrial transmembrane potential (ΔΨm) were defined in HCT116 and H460 cells. We did not observe DNA degradation and changes in the nuclei of normal cells, therefore, we did not perform further experiments concerning CCD 841 CoN and MRC-5 cells.

**Figure 7:**
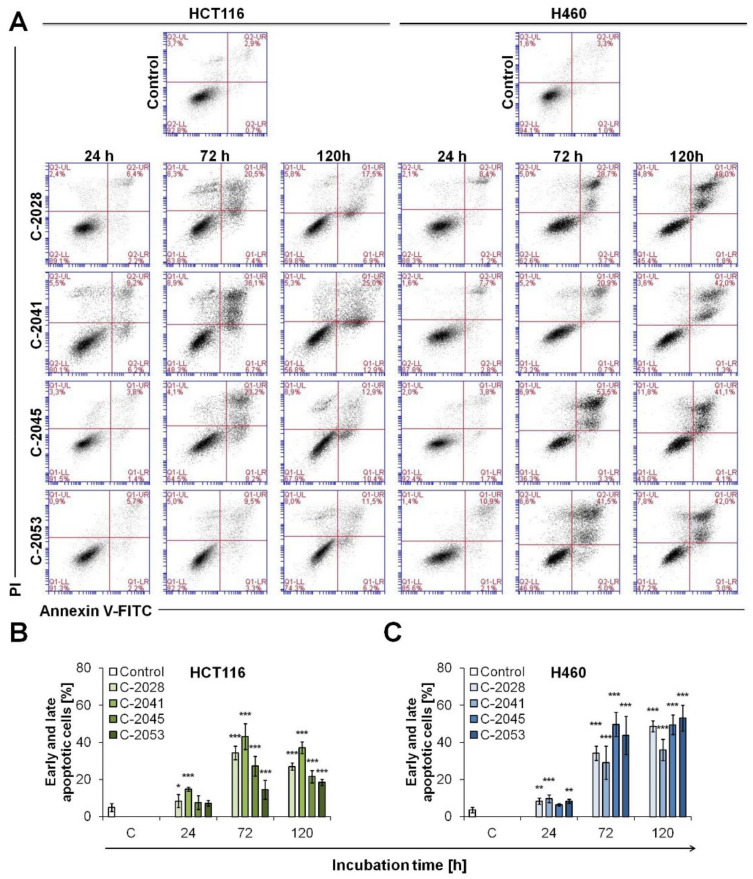
Phosphatidylserine externalization and membrane disruption in HCT116 and H460 cells treated with UAs. Cells were exposed to 0.04, 0.05, 0.4 and 0.2 µM of C-2028, C-2041, C-2045 and C-2053 compounds, respectively, stained with annexin V–fluorescein isothiocyanate (FITC) and propidium iodide (PI), and flow cytometry analysis was conducted. (**A**) Representative bivariate flow cytometry histograms of annexin V–FITC signal versus PI signal are shown. Bottom left quadrant represents living cells (annexin V negative, PI negative); bottom right quadrant represents early apoptotic cells (annexin V positive, PI negative); top right quadrant represents late apoptotic cells (annexin V positive, PI positive); top left quadrant represents primary necrotic cells (annexin V negative, PI positive). The cytograms shown are representative of at least three independent experiments. (**B**,**C**) Bar graphs show quantified data, expressed as the percentages of cells stained with annexin V–FITC alone or with PI—sum of early and late apoptotic cells established for HCT116 cell line (**B**) and H460 (**C**). Significantly different from the control at: * *p* < 0.05; ** *p* < 0.01; *** *p* < 0.001; (*n* ≥ 3).

All four bisacridine derivatives caused increasing changes in asymmetry and integrity of the membrane in HCT116 cells (Figure 7A). In C-2041 treated cells, the first marked alterations appeared as early as 24 h after drug exposure—a population of cells stained with annexin V alone or with propidium iodide (PI) arose. Most profound changes in asymmetry and integrity of the membrane were observed after 72 h of incubation with C-2041 and C-2045, which decreased slightly after 120 h. Annexin V positive cells (early and late apoptotic) accounted for approximately 43 and 27.8% of the total cell population treated for 120 h with C-2041 and C-2045, respectively. After 120 h, apoptosis (early and late) was observed in 26.1 and only 18.4% of cells exposed to C-2028 and C-2053, respectively (Figure 7B). Concomitantly, in all UAs treated HCT116 cells, necrosis was induced in a small percentage of the cells throughout the incubation period (cells stained only with PI). In summary, phosphatidylserine translocation, which is a very distinctive feature of apoptosis induction, in UAs treated HCT116 cells was most profound in cells exposed to C-2041 and least profound in cells exposed to C-2053.

Additionally, in H460 cells, many alterations in the cellular membrane were observed after UAs treatment (Figure 7C). During the time of incubation, the amount of early and late apoptotic cells increased. The most profound changes were observed after 120 h of incubation with the tested compounds, when the fraction of annexin V positive cells reached 48.7, 35.9, 49.4 and 53.1% for C-2028, C-2041, C-2045 and C-2053, respectively. A certain percentage of necrotic cells was observed after exposure to UAs, the highest in cells treated with C-2045 derivative—after 120 h, the amount of A^−^PI^+^ cells reached almost 12%. For the remaining three UAs, namely C-2028, C-2041 and C-2053, the amount of cells stained only with PI increased slightly during the incubation time, but it did not exceed 10%.

**Figure 8:**
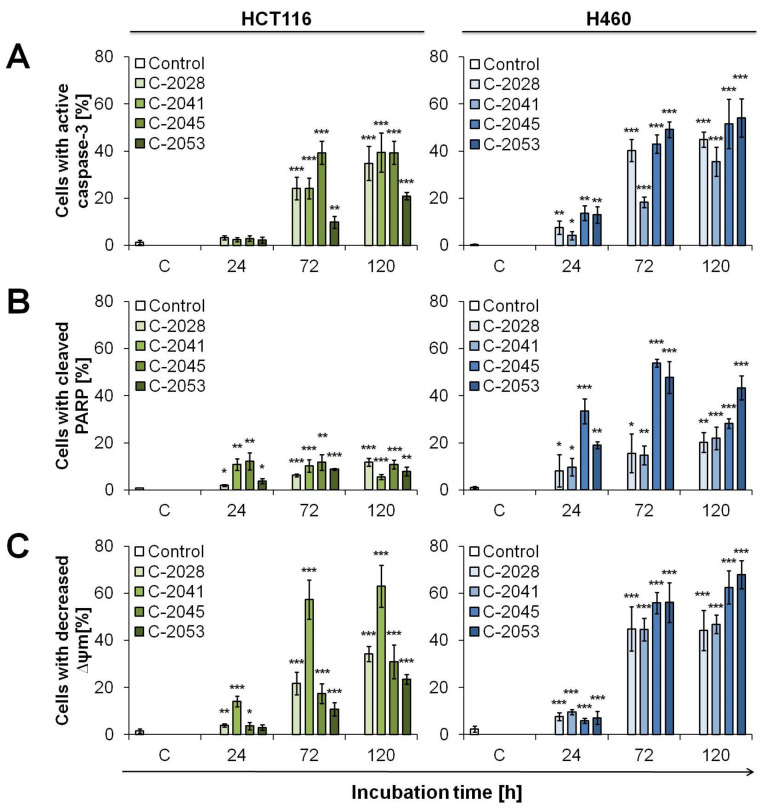
Cellular response in HCT116 and H460 cells induced by UA compounds. Cells were exposed to 0.04, 0.05, 0.4 and 0.2 µM of C-2028, C-2041, C-2045 and C-2053 compounds, respectively, for the times indicated and then subjected to appropriate staining and flow cytometry analysis described in detail in the Materials and Methods section. Bar graphs show quantified data, expressed as the percentages of cells with (**A**) active caspase-3, (**B**) cleaved PARP and (**C**) decreased mitochondrial transmembrane potential (ΔΨm). Significantly different from the control at: * *p* < 0.05; ** *p* < 0.01; *** *p* < 0.001; (*n* ≥ 3).

Another typical process for apoptosis is the activation of caspase-3, which cleaves PARP. Cytometric analysis of the presence of active caspase-3 revealed that the protein occurred significantly after 72 h of incubation with UAs, and after 120 h amounted to 34.8, 39.5, 39.3 and 20.9% for HCT116 cells exposed to C-2028, C-2041, C-2045 and C-2053 derivatives, respectively (Figure 8A). The activation of caspase-3 in H460 cells started already after 24 h of incubation with bisacridines. As the duration of treatment with UAs was extended, the number of lung cells with active caspase-3 gradually increased, and after 120 h of exposure, it reached 44.9, 35.5, 51.6 and 54.1% for C-2028, C-2041, C-2045 and C-2053, respectively (Figure 8A). PARP cleavage by caspase-3 in HCT116 cells remained at a relatively low level and did not exceed 12% in all UAs treated cells (Figure 8B). Moreover, PARP was least degraded in cells incubated with C-2053. Interestingly, the activation of caspase-3 and PARP cleavage did not occur to the greatest extent in HCT116 cells exposed to C-2041, unlike the processes of DNA degradation and annexin V externalization, but it appeared at a similar level as in cells treated with C-2045. Additionally, the cleavage of PARP protein occurred to the greatest extent in H460 cells treated with C-2045 and C-2053 derivatives (Figure 8B). The highest fraction of cells with cleaved PARP after treatment with these compounds was observed after 72 h, when it reached 53.9 and 47.8% for C-2045 and C-2053, respectively. Interestingly, after 120 h of treatment, this fraction decreased and constituted 28.2 for C-2045 and 43.3% for C-2053. The number of H460 cells with the degraded form of PARP increased throughout the incubation with C-2028 and C-2041 derivative, and after 120 h, it amounted to about 20%. 

Next, the decrease in mitochondrial transmembrane potential (ΔΨm), which is an early event in the process of apoptosis, was also evaluated (Figure 8C). In all UAs treated HCT116 cells, a systematic increase in the population of cells with a reduced ΔΨm was observed during the time of incubation, and after 120 h of exposure, it reached 34.3, 63.0, 30.9 and 23.5% for C-2028, C-1041, C-2045 and C-2053, respectively. Again, C-2041 turned out to be the most active derivative against HCT116 cells, while C-2053, the weakest. All four UA derivatives, along with the increasing time of treatment, caused more profound changes in the mitochondrial potential of H460 cells, and the amount of cells with decreased ΔΨm after 120 h of treatment with C-2028, C-2041, C-2045 and C-2053 was 44.3, 46.8, 62.5 and 67.8%, respectively (Figure 8C). Therefore, once again, the most profound alterations were observed in H460 cells after exposure to C-2045 and C-2053 compounds. 

### 2.7. UAs Induction of Accelerated Senescence in Cancer Cell Lines 

**Figure 9:**
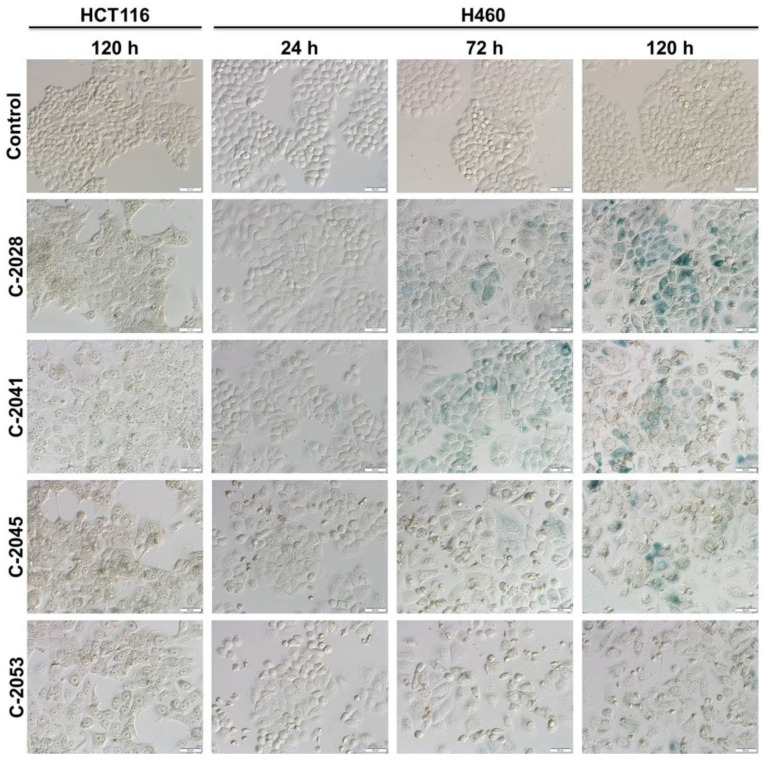
Accelerated senescence induction in HCT116 and H460 cells exposed to UAs derivatives. Representative images of cells examined for senescence associated with β-galactosidase (SA–β–gal) activity are shown. Cells were treated with 0.04, 0.05, 0.4 and 0.2 µM of C-2028, C-2041, C-2045 and C-2053 compounds, respectively, fixed, stained with X-gal, substrate for SA–β–gal enzyme, and analyzed under a bright-field inverted microscope (magnification ×200). The flattened and enlarged blue-colored cells were considered to be undergoing senescence. White scale bars presented on images correspond to 50 µm (*n* = 3).

Since cellular death was not induced in the entire population of tested cancer cells, in the next step we examined whether HCT116 and H460 cells underwent accelerated senescence after UAs exposure. SA-β-galactosidase (SA-β-gal) is an enzyme, which has been widely used as a marker of senescence. After incubation with X-gal (a substrate for SA-β-gal), in cells undergoing cellular senescence, a characteristic blue color appears, which results from the metabolic activity of the enzyme. This blue staining was observed in H460 cells after 72 and 120 h of incubation with four tested UAs, and the number of senescent cells increased during the incubation (Figure 9). Moreover, other features typical of senescent cells were observed in H460 cells, such as enlarged size, flattened shape and the appearance of granulations. The least profound changes were observed in the case of H460 cells incubated with C-2053 derivative, while C-2028 compound induced senescence to the highest extent. Interestingly, none of the studied UAs caused visible alterations in HCT116 cells, even after 120 h of incubation. Only in the case of C-2041, a few cells with characteristic blue color were observed (Figure 9).

### 2.8. The Ability of Cancer Cells to Return to Proliferation after UAs Treatment

**Figure 10:**
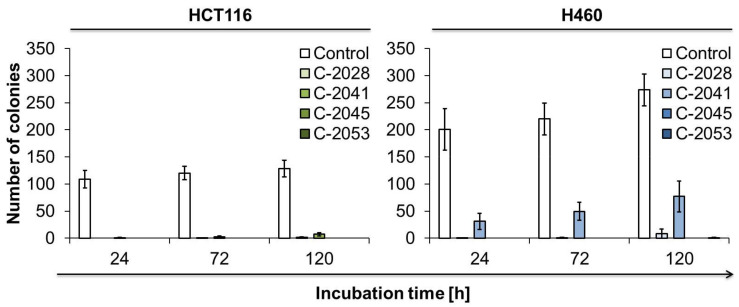
The ability of HCT116 and H460 cells to return to proliferation after UAs exposure. Cells were treated with 0.04, 0.05, 0.4 and 0.2 µM of C-2028, C-2041, C-2045 and C-2053 compounds, respectively. After the indicated drug exposure, approximately 250 cells were cultured for two weeks in fresh medium, and the number of colonies was counted. Bar graphs show quantified data, expressed as the number of colonies (*n* ≥ 3).

In order to determine whether HCT116 and H460 cells were able to return to proliferation after UAs exposure, a colony-forming assay was performed. After treatment with the studied compounds, approximately 250 cells were incubated for two weeks in fresh culture medium, and their ability to form colonies was observed (Figure 10). The exposure of HCT116 and H460 cells to C-2028 derivative resulted in an incomplete inhibition of cells proliferation—after 120 h of incubation, on average one and eight colonies were observed for HCT116 and H460 cells, respectively. Cell division was blocked to the least extent by C-2041 compound—in both HCT116 and H460 cells, the highest number of colonies was observed after 120 h of treatment, and on average 7 HCT116 cells and 77 H460 cells were able to undergo mitosis and divide, forming colonies. Importantly, the exposure of HCT116 and H460 cells to C-2045 and C-2053 completely blocked their return to proliferation, and this inhibition was observed already after 24 h of incubation with these compounds.

## 3. Discussion

Finding an effective and safe anticancer drug is still a demanding challenge. In our Department, we have developed a new group of compounds, unsymmetrical bisacridines (UAs), which exhibited high cytotoxicity against numerous human cancer cells and antitumor activity against several xenografts [3]. Additionally, UAs turned out to be very potent in inhibiting the growth of HCT116- and H460-derived spheroids [27]. Moreover, some of the UAs were conjugated with quantum dots (QDs), which improved their delivery to cancer cells [28,29,30]. In the presented study, we wanted to show the cellular mechanism of action of four UA derivatives in relation to changes in c-Myc and K-Ras proteins. We evaluated the influence of bisacridine compounds on cytotoxicity, expression and protein level of c-Myc and K-Ras, cell cycle progression and cellular response triggered in HCT116 colon and H460 lung cancer cells, as well as in normal cells—human colon CCD 841 CoN and lung MRC-5 cells. 

All tested UA derivatives exhibited high cytotoxicity against HCT116 and H460 cells, and IC_90_ doses did not exceed 0.4 µM. The calculated inhibitory doses were much lower than those, which are characteristic of clinically used drugs, such as irinotecan (colon cancer treatment) or cisplatin (lung cancer treatment). Their IC_50_ doses established in similar conditions are around 5 µM [31,32,33,34]. Most importantly, UAs cytotoxicity against normal cells turned out to be much lower than against cancer cells, especially for C-2045 and C-2053 compounds. The IC_90_ doses calculated for UA compounds against normal cells ranged between 2.7 and 6.2 µM, thus normal cells were much less sensitive to the tested drugs. This suggests that during patient treatment, normal cells may be less affected by chemotherapy using UAs than cancer cells. Furthermore, the analysis of the distribution of CCD 841 CoN and MRC-5 cells in the cell cycle showed no significant changes between control cells and those treated with UAs for 120 h at the same concentration as cancer cells. Only up to 6% of UAs-exposed normal cells had degraded DNA. Microscopic observation of nucleus morphology also did not reveal induction of cell death in CCD 841 CoN and MRC-5 cells incubated with bisacridines. In summary, at a concentration that strongly inhibited the proliferation of HCT116 and H460 cancer cells and induced cell death, normal cells, CCD 841 CoN and MRC-5, remained almost unaffected, which makes UAs very clinically attractive drugs. 

The unique property, which UA compounds possess, is stabilization of the four-stranded nucleic acids structures, known as G-quadruplex (G4) of DNA [3]. G4 structures are present in the promoter regions of regulatory factors and oncogenes controlling cellular proliferation, such as *MYC*, *KIT*, *RAS* genes, *BRAF*, *BCL2*, *HIF*, *VEGF*, *HSP90*, *MET*, *RET*, androgen receptor [35], in telomeric repeats and immunoglobulin switch regions [36,37]. Various studies have revealed that the G4 motif is over-represented in promoter regions of oncogenes and apparently almost absent in tumor suppressor genes. Thus, this suggests that G4 structures are a valuable target for anticancer therapy [37]. G4 can also be found in mRNA of the following proteins: FMRP, FGF-β, N-Ras, Zic-1, IGF-II and HIV-1 genomic RNA [4]. Moreover, G4 structures are characteristic of 5′ untranslated regions (UTR), which take part in the regulation of the translation process [38]. *BCL2* and *NRAS* mRNA have been shown to contain G4 structures in the UTR regions, which may affect the translation of these genes [39,40]. Our previous studies have shown that some of the UA derivatives, including those used in this study, can stabilize promoter regions of *MYC* and *KRAS* genes. These findings were determined in an in vitro assay based on the measurement of the melting points of oligonucleotides containing FAM and TAMRA dyes [3]. Therefore, we decided to analyze the influence of our drugs on the expression and protein level of c-Myc and K-Ras in cancer and normal cell lines, HCT116, H460, CCD 841 CoN and MRC-5. *KRAS* mRNA level in HCT116 cells remained the same during treatment with UAs, while in H460, a small increase during the time of incubation was observed. Furthermore, the protein level of K-Ras in both cell lines increased slightly after prolonged exposure time. Thus, our UA compounds did not affect K-Ras, and the observed slight upregulation may be due to its natural function in cell signaling pathway in cancer cells [41], especially since HCT116 and H460 cells have a mutant protein [42]. In normal CCD 841 CoN and MRC-5 cells, the expression and protein level of K-Ras did not change. However, finding a strong and effective inhibitor of K-Ras is an enormous challenge [43]. Attempts are also being made to find molecules that target the G4 structures present in the promoter region of *KRAS* [44]. 

The influence of UA derivatives on the expression and protein level of c-Myc turned out to be much more complicated. In HCT116 cells, the mRNA level of *MYC* increased several times after 72 h of incubation compared to untreated control cells. However, the c-Myc protein level did not change during the time of incubation, suggesting that the UAs altered the translation process of c-Myc and restored its normal expression. Interestingly, in H460 cells, UAs caused a slight decrease in *MYC* expression, especially in the case of C-2045 and C-2053 drugs, where the level of *MYC* mRNA dropped by 50%. Moreover, UA compounds also strongly affected c-Myc translation, and after 72 h of treatment, the c-Myc protein almost disappeared. There are more and more examples of compounds that can target the c-Myc protein [10,45]. UA derivatives may be among them. There are also compounds that can trigger both expression and translation of specific proteins with G4 structures present in their DNA and mRNA [39,46]. It has been shown that in various cell lines, the possible interaction of the tested compound with G4 structures may occur with different effectiveness, from very weak to strong [35,47,48]. This may explain the differences in the interactions of UA compounds with c-Myc mRNA and protein between HCT116 and H460 cells. Further investigation has to be undertaken to explain this phenomenon.

The strong influence of UA compounds on c-Myc protein seems to have an impact on cellular death and senescence in the studied cancer cell lines. The main type of cell death induced in HCT116 and H460 cells was apoptosis. In lung cancer cells, where c-Myc was inhibited, apoptosis was induced to a greater extent. All conducted experiments, such as the morphological observation of cell nuclei, externalization of phosphatidylserine, membrane permeability, caspase-3 activation, PARP cleavage and mitochondrial potential decrease proved that starting from 72 h of exposure, strong induction of apoptosis occurred in these cells. The first signs of cell death, such as caspase-3 activation and PARP cleavage, appeared even after 24 h in H460 cells exposed to C-2045 and C-2053 compounds. These two derivatives turned out to be the most effective against lung cancer cells and, after 120 h of incubation, about 60% of treated cells underwent apoptosis. C-2041 turned out to be the weakest compound, especially when considering caspase-3 activation and annexin V binding. Importantly, MYC expression increased only in C-2041-H460 treated cells. Interestingly, in the case of HCT116 colon cancer cells, C-2041 was the compound that affected the cells the most. DNA degradation and mitochondrial potential decrease were observed in around 70% of colon cells exposed to C-2041, while in C-2028 and C-2045 treated cells, only in 30%. In addition, processes specific to apoptosis, such as caspase-3 activation and PARP cleavage, occurred in cells incubated with C-2041 to a similar extent as in those treated with C-2028 and C-2045 (40% and approximately 10% of cells with active caspase-3 and cleaved PARP, respectively), which is significantly weaker. These observations suggest that C-2041 differed in its way of cellular action from other UAs and that cells treated with this derivative underwent more nonspecific cell death. Here, it is worth to mention that in studies concerning the influence of UAs on 3D spheroids cultures, C-2041 was the compound that inhibited the growth of both HCT116 and H460 spheroids to the least extent [27]. Another noteworthy observation is that C-2053 was the derivative, which was the least active against HCT116 colon cells, but one of the most potent ones against H460 lung cancer cells. Moreover, in 3D culture, C-2053 inhibited the growth of spheroids to the greatest extent, especially in the case of HCT116 cells [27]. The colony-forming assay also confirmed that C-2045 and C-2053 compounds were the most effective derivatives among UAs—cells of both HCT116 and H460 cell lines, after drug treatment, did not return to proliferation. C-2041, and to a small extent also C-2028, did not manage to completely block colon and lung cancer cell division, even though C-2041 induced apoptosis in HCT116 cells to the greatest extent.

The other cellular process, which differed between the two studied cancer cell lines, HCT116 and H460, was the induction of accelerated senescence by UAs. In H460 cells, where c-Myc was completely inhibited by all bisacridines, senescence was observed in all exposed cells. C-2028 triggered senescence the most, while C-2053 the least. In HCT116 cells, the characteristic blue staining was only present in a few C-2041 treated cells. It is well known that cells in which c-Myc is downregulated are more prone to accelerated senescence [12]. Moreover, compounds, which are described as c-Myc inhibitors, usually induce senescence in a larger population of treated cells [49,50]. 

It is worth to mention that the derivatives of UA were developed based on the combined chemical structures of two compounds previously synthesized in our Department—C-1311 [51] and C-1748 [52]. C-1311 reached phase II clinical trials [53] and has been extensively investigated in various cell lines. Studies have shown that C-1311 is a DNA intercalator and topoisomerase II inhibitor [54]. It caused the accumulation of cells in the G2/M phase and subsequent induction of apoptosis, necrosis, autophagy, mitotic catastrophe or accelerated senescence, wherein apoptosis was not the main cause of cell death in solid cancer cell lines, especially HCT116 [55] and H460 cells [56]. Therefore, it should be emphasized that the presented studies showed that UAs exhibit an entirely different cellular mechanism of action to C-1311.

In summary, bisacridine derivatives strongly affected the expression and translation of c-Myc in the HCT116 and H460 cancer cell lines. Downregulation of the c-Myc protein level resulted in the induction of apoptosis and accelerated senescence to a greater extent in lung cancer cells treated with UA derivatives, especially C-2045 and C-2053. A possible molecular explanation for such a phenomenon could be the interaction of UAs with G4 structures present in the promoter region of the *MYC* gene or its mRNA. Further research should be undertaken to illustrate this relationship. UA compounds are examples of molecules that can inhibit c-Myc in some cancer cells and may, therefore, be used as therapeutic agents in anticancer therapies. 

## 4. Materials and Methods

### 4.1. Chemicals and Reagents

Unsymmetrical bisacridine derivatives (UAs), C-2028, C-2041, C-2045 and C-2053, were synthesized in the Department of Pharmaceutical Technology and Biochemistry, Gdańsk University of Technology, according to a previously published procedure [3]. Three of them, C-2028, C-2041 and C-2045, were prepared as methanosulphonians and the last one, C-2053, as monochloride, to achieve the best solubility and stability. Stock and working solutions of all drugs were prepared in sterile and deionized water (Milli-Q, Merck, Darmstadt, Germany).

RIPA buffer and anti-K-Ras antibody were purchased from Abcam (Cambridge, UK). Anti-c-Myc antibody and secondary anti-mouse and anti-rabbit horseradish peroxidase-linked antibody were purchased from Cell Signalling Technology (Beverly, MA, USA). Anti-β-actin antibody was ordered from Sigma-Aldrich (St. Louis, CA, USA). PI/RNase Staining Buffer, FITC Annexin V Apoptosis Detection Kit, Mitochondrial Membrane Potential Detection JC-1 Kit, Alexa Fluor 647 Rabbit anti-Active Caspase-3 antibody, Alexa Fluor 647 Mouse anti-Cleaved PARP antibody and Fixation/Permeabilization Solution Kit were obtained from BD Biosciences (San Diego, CA, USA). Fetal bovine serum (FBS) was purchased from Biowest (Nuaille, France). Maxima Hot Start Green PCR Master Mix and GeneRuler 50 bp DNA Ladder were obtained from Thermo Scientific (Waltham, MA, USA). High Pure RNA Isolation Kit, Transcriptor First Strand cDNA Synthesis Kit, The LightCycler^®^ 480 SYBR Green I Master, Phosphatase Inhibitor Cocktail Tablets PhosSTOP^TM^ and Mini EDTA-free Protease Inhibitor Cocktail cOmplete^TM^ were purchased from Roche (Manheim, Germany). Primers for PCR reaction were ordered from Genomed (Warsaw, Poland). Protein Assay Dye Reagent Concentrate and DC Protein Assay were obtained from Bio-Rad (Hercules, CA, USA). The following reagents were purchased from Merck-Sigma-Aldrich (Darmstadt, Germany): 3-(4,5-dimethylthiazol-2-yl)-2,5-diphenyltetrazolium bromide (MTT), agarose, ammonium persulfate, crystal violet solution, dimethyl sulfoxide (DMSO), ethidium bromide, ethylenediaminetetraacetic acid (EDTA), formaldehyde, glutaraldehyde, glycine, magnesium chloride, Hoechst 33342, McCoy’s 5A medium, MEM medium, penicillin-streptomycin solution, phenylmethanesulfonyl fluoride (PMSF), potassium chloride, potassium ferrocyanide, potassium ferricyanide, potassium phosphate dibasic, potassium phosphate monobasic, sodium deoxycholate, sodium dodecyl sulfate (SDS), sodium phosphate, sodium phosphate dibasic, RPMI 1640 medium, Tris base, Tris hydrochloride, Trypsin-EDTA solution, Tween 20, X-gal (5-bromo-4-chloro-3-indolyl-β-*D*-galactosidase). The following reagents were purchased from POCH S.A. (Gliwice, Poland): ethanol, methanol, sodium chloride, sodium fluoride, acetic acid. Ultra-pure deionized water R > 18 MΩ cm-1 was prepared by Milli-Q Integral Water Purification System, Merck Millipore (Billerica, MA, USA).

### 4.2. Cell Lines and Culture

In the presented project, four cell lines were used: two carcinoma, HCT116 and H460, and two normal, CCD 841 CoN and MRC-5. All cell lines were purchased from the American Type Culture Collection (Manassas, VA, ATCC) and were tested negatively for mycoplasma using the Universal Mycoplasma Detection Kit (ATCC). Colorectal carcinoma HCT116 cells were maintained in McCoy’s 5A medium (Merck/Sigma-Aldrich, Darmstadt, Germany), and non-small-cell lung carcinoma H460 cells were maintained in RPMI 1640 medium (Merck/Sigma-Aldrich, Germany). Both media were supplemented with 10% fetal bovine serum (FBS; Biowest, Riverside, MO, USA), 100 μg/mL streptomycin and 100 unit/mL of penicillin. Human colon epithelial CCD 841 CoN cells and human lung fibroblast MRC-5 cells were maintained in Eagle’s Minimal Essential Medium (MEM; Merck/Sigma-Aldrich) supplemented with 10% FBS without antibiotics. All cells were incubated in 5% CO_2_ atmosphere at 37 °C. The experiments were performed with cells in the exponential phase of growth.

### 4.3. Cell Growth Inhibition Assay

To assess cell viability, the MTT assay was used. HCT116, H460, CCD 841 CoN and MRC-5 cells (20,000/well and 50,000/well for cancer and normal cells, respectively) were seeded in 24-well plates, and the following UAs were added at concentrations up to 10 μM: C-2028, C-2041, C-2045 and C-2053. Stock solutions (10 mM) were prepared in sterile water, as well as dilutions, also in water. After 72 h, 3-(4, 5-dimethylthiazol-2-yl)-2,5-diphenyltetrazolium bromide (MTT; 800 μg/well) was added for 3 h, then the formazan crystals were dissolved in DMSO, and absorbance was read at 540 nm. Each point was conducted at least 4 times, and data are expressed relative to controls. The concentration of the drug required to inhibit cells growth by 50 (IC_50_), 80 (IC_80_) and 90% (IC_90_) compared to untreated control cells was determined from the curves plotting survival as a function of dose. 

### 4.4. mRNA Isolation and Real-Time Quantitative Polymerase Chain Reaction (RT-PCR)

HCT116, H460, CCD 841 CoN and MRC-5 cells after treatment with UAs were collected, washed twice with ice-cold PBS and resuspended in 200 mL of PBS. mRNA was isolated using High Pure RNA Isolation Kit according to the manufacturer’s instructions (Roche, Basel, Switzerland). Briefly, cells were lysed in lysis-binding buffer and transferred to a filter tube. Contaminating DNA was removed by 15 min incubation of the filter tube with DNase I solution. The filter tube was washed once with buffer I and twice with buffer II, and after centrifugation, total RNA was eluted using the elution buffer. RNA concentration was determined by NanoDrop One (Thermo Scientific); 1 µg of RNA was reverse transcribed using Transcriptor First Strand cDNA Synthesis Kit according to the manufacturer’s instructions (Roche) in 20 µL of reaction containing 1 µg of RNA, 2.5 mM anchored-oligo(dT)_18_Primer, 8 mM MgCl_2_, 20 U of Protector RNase Inhibitor, deoxynucleotide mix (1 mM each) and 22 U of Transcriptor Reverse Transcriptase. The reverse transcription reaction was carried out for 30 min at 55 °C and stopped by heating to 85 °C for 5 min and placing on ice. The cDNA solution was stored at −20 °C until use. Real-time polymerase chain reaction (PCR) was performed on 2 µL of cDNA using 0.2 µM forward and reverse primers on LightCycler^®^ 480 using LightCycler^®^ 480 SYBR Green I Master according to manufacturer’s instructions (Roche). Primers were designed using BLAST website. Their efficiency was checked, and they were ordered from Genomed (Warsaw, Poland). The sequences of primers were as follows: *MYC* set 1 forward primer, CGTCCTCGGATTCTCTGCTC; *MYC* set 1 reverse primer, GCTGCGTAGTTGTGCTGATG; *MYC* set 2 forward primer, GCCTTGGTTCATCTGGGTCT; *MYC* set 2 reverse primer, TGCTTAGGAGTGCTTGGGAC; *KRAS* forward primer, ATCTTCAGTGCCAGTCTTGGG; *KRAS* reverse primer, TCAAGTCATGGGGCATGTGG; reference gene, *GAPDH* forward primer ACCCACTCCTCCACCTTTG; and *GAPDH* reverse primer, CTCTTGTGCTCTTGCTGGG. Cycling conditions were as follows: 1 cycle for 7 min at 95 °C (preincubation), 45 cycles of amplification: 10 s at 95 °C (denaturation), 20 s at 62 °C (annealing), 30 s at 72 °C (extension), 1 cycle for obtaining melting curve and 1 cycle for 10 s at 40 °C (cooling). The results were standardized to the reference gene *GADPH*. The relative expression level of *MYC* and *KRAS* was quantified using comparative method 2^ΔΔCt^ [26]. The results were obtained from three independent experiments, each of which was analyzed at least twice.

### 4.5. Western Blotting

HCT116, H460, CCD 841 CoN and MRC-5 cells were scraped, pooled with floating cells and washed twice with ice-cold PBS. Cells were suspended in RIPA buffer (Abcam, Cambridge, Great Britain) with protease and phosphatase inhibitor cocktail (Roche), 1 mM phenylmethanesulfonyl fluoride (PMSF) and kept for 20 min on ice with brief vortexing every 5 min. Lysates were then centrifuged at 14,000 g for 15 min at 4 °C. The protein concentration was determined using the DC Protein Assay (Bio-Rad, Hercules, CA, USA). Samples were mixed with Laemmli buffer (Bio-Rad) and denatured at 100 °C for 5 min. Then, 30 μg of total protein was subjected to SDS-PAGE and transferred onto nitrocellulose membranes using a semi-dry blotting apparatus (Bio-Rad, Hercules, CA, USA). The membrane was blocked with 5% non-fat milk in TBST buffer, rinsed five times with TBST buffer and probed with primary antibodies. Mouse anti-c-Myc was purchased from Cell Signaling Technology (Beverly, MA, USA). Rabbit anti-K-Ras antibody was purchased from Abcam (Cambridge, Great Britain) or Santa Cruz Biotechnology (Dallas, TX, USA). Anti-β-actin antibody was ordered from Sigma-Aldrich (St. Louis, CA, USA). Secondary anti-mouse and anti-rabbit horseradish peroxidase-linked antibody was purchased from Cell Signaling Technology. For each Western blot, chemiluminescence detection was performed using enhanced chemiluminescence system reagents (Thermo Scientific, Waltham, MA, USA). Densitometry analysis was performed using ImageJ Software (U. S. National Institutes of Health, Bethesda, MD, USA).

### 4.6. Cell Cycle Analysis

HCT116, H460, CCD 841 CoN and MRC-5 cells were exposed to UAs at IC_90_ doses for 24 to 120 h. After drug treatment, trypsinized and floating cells (2 × 10^6^) were pooled, washed twice with ice-cold PBS and fixed in 70% (*v*/*v*) ethanol overnight at −20 °C. Cells were stained with propidium iodide (PI) (BD Biosciences, San Jose, CA, USA) and monitored by FACS Accuri C6 (BD Biosciences, San Jose, CA, USA).

### 4.7. Assessment of Cell Morphology

Nuclear morphology was examined under a fluorescence microscope (OLYMPUS BX60, 400× objective lens) after staining with Hoechst 33342. Briefly, after drug treatment, HCT116, H460, CCD 841 CoN and MRC-5 cells were spun onto microscopic slides, fixed with methanol:acetic acid (3:1) for 15 min and stained with Hoechst 33342 (1 mg/mL) for 15 min. Cells were regarded as apoptotic based on the presence of condensed, fragmented chromatin. Enlarged cells containing multiple nuclei were considered as typical of mitotic catastrophe.

### 4.8. Annexin V/PI Dual Staining

Annexin V–FITC binding was performed with an Annexin-V Fluos Staining kit (BD Biosciences, San Jose, CA, USA) according to manufacturer’s protocol. Briefly, after drug treatment, HCT116 and H460 cells (1 × 10^6^) were trypsinized, washed twice with ice-cold PBS, pelleted and resuspended in 100 µL of binding buffer containing Annexin-V–FITC and PI. Cells were incubated for 15 min at room temperature in the dark, then diluted with 400 µL of binding buffer and analyzed by flow cytometry with FACS Accuri C6 (BD, San Jose, CA, USA). Cells with low FITC and PI fluorescence were considered viable. Cells that presented high FITC fluorescence but low PI fluorescence were regarded as early apoptotic. Late apoptotic cells presented high FITC and PI fluorescence. Necrotic cells had low FITC fluorescence and high PI fluorescence.

### 4.9. Caspase-3 Activity Measurement

Caspase-3 activity was determined using Alexa Fluor 647-conjugated anti-active caspase-3 antibody (BD Biosciences, San Diego, CA, USA) according to the manufacturer’s instructions. Briefly, HCT116 and H460 cells (1 × 10^6^) were trypsinized, pelleted, washed twice with ice-cold PBS and fixed in Fixation Buffer for 20 min on ice. After fixation, cells were rinsed twice with Perm Wash Buffer, stained with Alexa Fluor 647-conjugated anti-active caspase-3 antibody for 30 min, rinsed with Perm Wash Buffer and analyzed by flow cytometry with FACS Accuri C6 (BD, San Jose, CA, USA).

### 4.10. PARP Cleavage Analysis

PARP cleavage was determined using Alexa Fluor 647-conjugated anti-cleaved PARP antibody (BD Biosciences, San Diego, CA, USA) according to the manufacturer’s instructions. Briefly, HCT116 and H460 cells (1 × 10^6^) were trypsinized, pelleted, washed twice with ice-cold PBS and fixed in Fixation Buffer for 20 min on ice. After fixation, cells were rinsed twice with Perm Wash Buffer, stained with Alexa Fluor 647-conjugated anti-cleaved PARP antibody for 30 min, rinsed with Perm Wash Buffer and analyzed by flow cytometry with FACS Accuri C6 (BD, San Jose, CA, USA).

### 4.11. Mitochondrial Transmembrane Potential Measurements

Changes in the mitochondrial membrane potential (ΔΨm) were analyzed by flow cytometry using 5,5′,6,6′-tetrachloro-1,1′,3,3′-tetraethylbenzimidazolyl-carbocyanine iodide, JC-1 (BD Biosciences, San Jose, CA, USA) according to manufacturer’s specification. Briefly, after treatment, HCT116 and H460 cells (1 × 10^6^) were trypsinized, pelleted, resuspended in media and stained with JC-1 for 15 min at 37 °C. After staining, cells were washed twice with assay buffer and analyzed by flow cytometry with FACS Accuri C6 (BD, San Jose, CA, USA). The loss of ΔΨm was monitored based on the decrease in JC-1 red fluorescence concurrent with the increase in green fluorescence. 

### 4.12. Accelerated Senescence Observations

The accelerated senescence examination was based on the analysis of pH 6.0-dependent β-galactosidase (SA-β-gal) expression together with senescence-related morphology, such as enlarged and flattened cells. HCT116 and H460 cells were seeded in 60 mm plates containing microscope coverslips and treated with UAs for up to 120 h. After the treatment, coverslips with attached cells were moved to 35 mm dishes, washed twice with PBS and fixed with 2% glutaraldehyde and 0.2% formaldehyde for 5 min. The cells were then washed twice with PBS (pH 6.0) and incubated at pH 6.0 with X-gal (5-bromo-4-chloro-3-indolyl-β-*D*-galactosidase, substrate for SA-β-gal) staining solution (1 mg/mL X-gal, 40 mmol/L citric acid/sodium phosphate, pH 6.0, 5 mmol/L potassium ferrocyanide, 5 mmol/L potassium ferricyanide, 150 mmol/L NaCl, 2 mmol/L MgCl_2_). Following overnight incubation at 37 °C, cells were washed twice with PBS and observed under a light microscopy (OLYMPUS BX60; magnification ×200).

### 4.13. Colony-Forming Assay

The ability of cells to return to proliferation after UAs treatment was measured by a colony-forming assay. After drug treatment, 250 HCT116 and H460 cells were harvested and seeded in a new 6-well plate containing fresh medium without the drug and incubated for two weeks. Untreated cells were used as controls. After two weeks, colonies were fixed with 80% ethanol, stained with crystal violet, counted, and photos were taken.

### 4.14. Data Analysis 

All data presented represent the mean of the results from at least 3 independent experiments ± standard deviation. The statistical significance of the differences between the variables was determined using the unpaired Student’s *t*-test or one sample t and the Wilcoxon test (RT-PCR and Western blotting studies). The values were compared using statistical analysis performed with Graph–Pad Prism 5.0 (San Diego, CA, USA). * *p* < 0.05, ** *p* < 0.01 and *** *p* < 0.001 were considered significant.

## 5. Conclusions

Unsymmetrical bisacridines (UAs) are newly synthesized compounds with antitumor properties. They have been shown to stabilize G-quadruplex structures in the promoter region of some oncogenes, including K-Ras and c-Myc. In the presented study, we aimed to determine the cellular response triggered by UA derivatives with respect to changes in the c-Myc and K-Ras protein level. Bisacridine compounds exhibited high cytotoxicity against HCT116 colorectal and H460 lung cancer cells. Importantly, normal human cells, CCD 841 CoN colon and MRC-5 lung, were less sensitive to UAs, and the degradation of DNA and changes in nuclei morphology did not occur in these cells after prolonged incubation time. Furthermore, treatment of HCT116 and H460 cells with bisacridines did not influence K-Ras; however, it strongly affected the expression and protein level of c-Myc. As a results of these processes, c-Myc remained at the same level in HCT116 cells, while being completely inhibited in H460. Downregulation of the c-Myc protein level resulted in the induction of apoptosis and accelerated senescence to a greater extent in lung cancer cells treated with UA derivatives, especially C-2045 and C-2053. A possible molecular explanation for such a phenomenon may be the interaction of UAs with G4 structures present in the promoter region of the *MYC* gene or its mRNA.

Summing up, we showed that UA compounds at low concentrations induced apoptosis in HCT116 colorectal and H460 lung cancer cells, while having slight impact on normal cells. UAs can inhibit c-Myc protein in some cell types, enhancing their ability to undergo apoptosis and accelerated senescence, which makes UAs attractive therapeutic agents. 

## Figures and Tables

**Table 1:** Cytotoxicity of UAs against HCT116, H460, CCD 841 CoN and MRC-5 cells (*n* ≥ 4).

Compound	Drug Dose [µM]	Cell Line			
		HCT116	H460	CCD 841 CoN	MRC-5
**C-2028**	IC_50_	0.010 ± 0.001	0.016 ± 0.002	0.025 ± 0.007	0.020 ± 0.004
	IC_90_	0.044 ± 0.005	0.046 ± 0.004	3.69 ± 1.08	2.69 ± 0.64
**C-2041**	IC_50_	0.008 ± 0.002	0.015 ± 0.002	0.036 ± 0.005	0.023 ± 0.004
	IC_90_	0.049 ± 0.005	0.046 ± 0.005	5.12 ± 1.09	6.16 ± 2.06
**C-2045**	IC_50_	0.059 ± 0.008	0.095 ± 0.038	0.338 ± 0.027	0.218 ± 0.037
	IC_90_	0.455 ± 0.026	0.399 ± 0.052	5.34 ± 1.85	6.13 ± 2.35
**C-2053**	IC_50_	0.048 ± 0.014	0.025 ± 0.006	0.240 ± 0.040	0.120 ± 0.026
	IC_90_	0.195 ± 0.075	0.184 ± 0.022	4.10 ± 0.47	3.80 ± 1.34

## Data Availability

The data presented in this study are available on request from the corresponding author.

## Supplementary Materials

The following supporting information can be downloaded at: https://www.mdpi.com/article/10.3390/ijms23063061/s1.
