# Supplementary material for: c-Myc Protein Level Affected by Unsymmetrical Bisacridines Influences Apoptosis and Senescence Induced in HCT116 Colorectal and H460 Lung Cancer Cells
Source: Int J Mol Sci. 2022 Mar 11;23(6):3061. doi: 10.3390/ijms23063061 (PMC8955938; doi:10.3390/ijms23063061)
Supplement: Supplementary file 1 [file ijms-23-03061-s001.zip › ijms-1600296-supplementary.pdf]

International Journal of *Molecular Sciences*

MDPI

*Supplementary Data*

# c-Myc Protein Level Affected by Unsymmetrical Bisacridines Influences Apoptosis and Senescence Induced in HCT116 Colorectal and H460 Lung Cancer Cells

**Monika Pawłowska, Jolanta Kulesza and Ewa Augustin ***

Department of Pharmaceutical Technology and Biochemistry, Faculty of Chemistry, Gdańsk University of Technology, 80-233 Gdańsk, Poland; monpawlo@pg.edu.pl (M.P.); jolanta.kulesza@pg.edu.pl (J.K.)

* Correspondence: ewa.augustin@pg.edu.pl; Tel.: +48-58-347-14-68

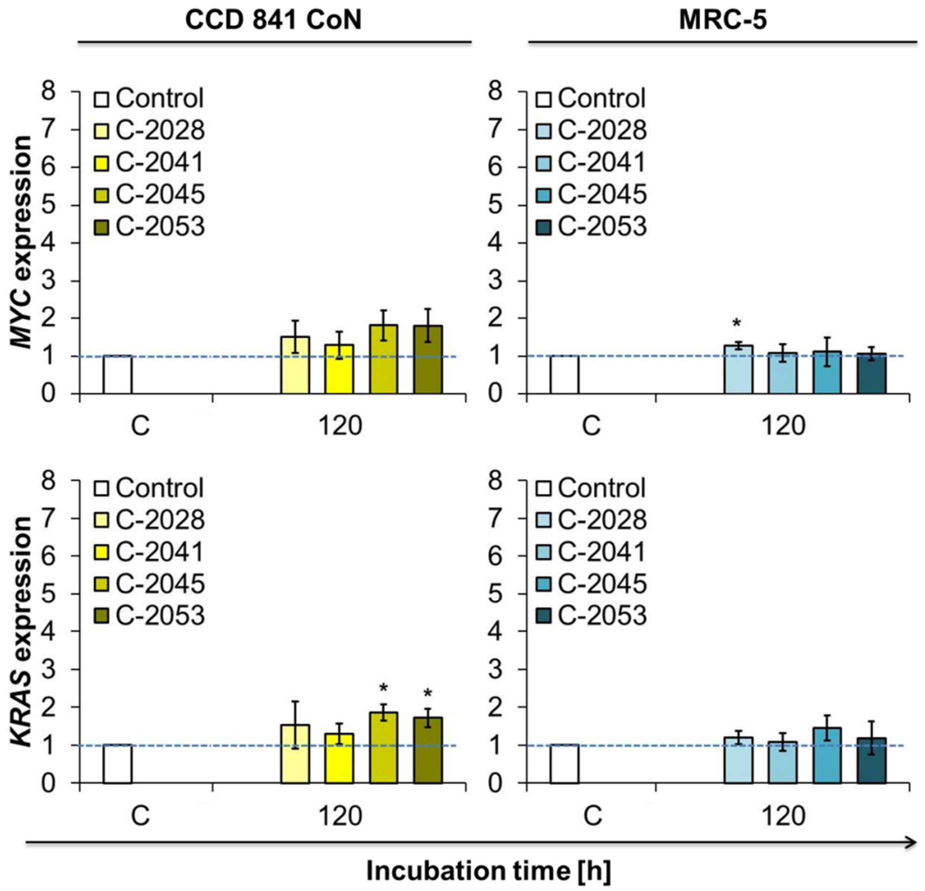


**Figure S1.** *MYC* and *KRAS* expression in CCD 841 CoN colon and MRC-5 lung normal cells. Cells were incubated with $IC_{90}$ doses of UAs for 120 h, total mRNA was isolated, transcribed to cDNA, and real-time PCR analysis was performed with the appropriate primers for the *MYC* and *KRAS* genes. *ACTB* (β-actin) was used as housekeeping gene standard. Relative gene expression was calculated using 2^-ΔΔCt method [Livak]. Significantly different from the control at: *$p < 0.05$; **$p < 0.01$; ***$p < 0.001$, n = 3).

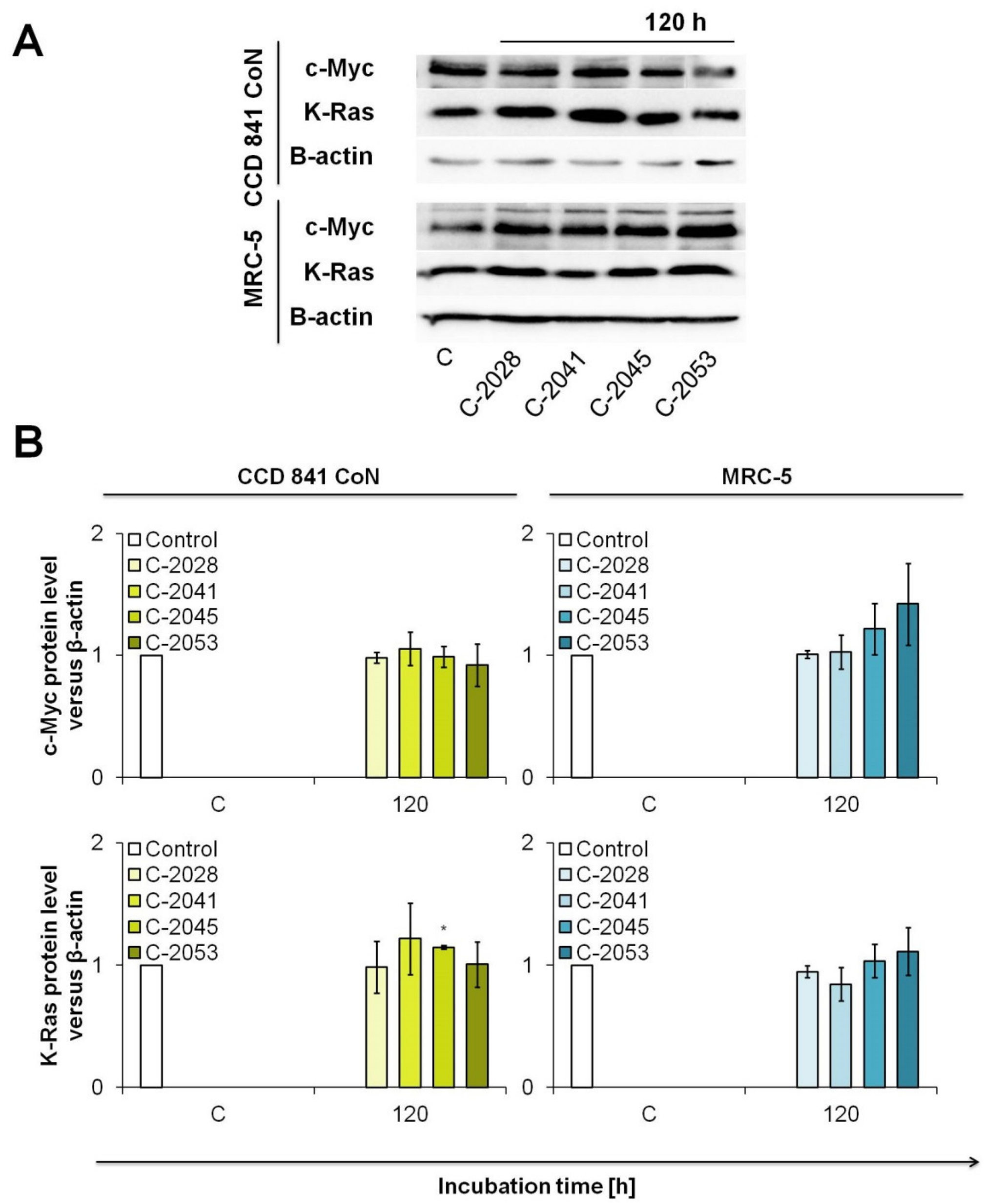


**Figure S2.** Western blot analysis of c-Myc and K-Ras protein levels in CCD841 CoN colon and MRC-5 lung normal cells. Cells were incubated with $IC_{90}$ doses of UAs for 120 h, whole cell extracts were prepared, 20 μg of proteins/lane were separated by poliacrylamide gel electrophoresis and semi-dry transferred on a membrane. Protein levels were detected after immunostaining the membrane with appropriate antibodies and ECL developing. (**A**) Representative Western blot analysis of c-Myc and K-Ras in CCD 841 CoN and MRC-5 cells and (**B**) their relative densitometry quantification performed using ImageJ Software. Significantly different from the control at: *$p < 0.05$; **$p < 0.01$; ***$p < 0.001$; (n = 3).

**Table S1.** Cell cycle distribution in UAs treated HCT116 cells. Flow cytometry analysis of cell cycle distribution of HCT116 untreated (control) and treated cells with C-2028, C-2041, C-2045 and C-2053 at concentrations corresponding to $IC_{90}$ doses for the times indicated. Data shows percentages of cells in the sub-G1, G1, S, G2/M, and Poli (polyploid cells) phases of the cell cycle. Data represents the averages of at least three independent experiments with standard deviation (n ≥ 3).

| | | **Phase of Cell Cycle** | | | | |
|---|---|---|---|---|---|---|
| **Compound** | **Incubation Time [h]** | **Sub-G1** | **G1** | **S** | **G2/M** | **Poli** |
| **Control** | **24** | 2.03 ± 0.43 | 48.54 ±2.58 | 16.72 ± 2.01 | 22.88 ± 2.27 | 9.98 ± 1.81 |
| **C-2028** | **24** | 3.37 ± 0.69 | 24.89 ± 3.90 | 19.33 ± 1.58 | 45.89 ± 3.13 | 5.73 ± 0.93 |
| | **72** | 36.97 ± 8.93 | 28.63 ± 5.96 | 7.67 ± 2.54 | 21.02 ± 4.86 | 5.11 ± 1.54 |
| | **120** | 37.46 ± 9.35 | 25.39 ± 3.25 | 4.95 ± 1.90 | 26.26 ± 4.90 | 5.73 ±2.33 |
| **C-2041** | **24** | 11.81 ± 1.94 | 28.75 ± 3.88 | 25.97 ± 2.80 | 29.92 ± 4.56 | 4.22 ± 2.00 |
| | **72** | 48.83 ± 7.71 | 22.16 ± 5.06 | 9.59 ± 2.24 | 14.29 ± 1.52 | 4.47 ± 2.13 |
| | **120** | 68.86 ± 9.48 | 13.63 ± 3.71 | 3.25 ± 1.05 | 9.44 ± 3.88 | 4.64 ± 2.56 |
| **C-2045** | **24** | 3.90 ± 0.83 | 24.61 ± 2.63 | 16.33 ± 1.34 | 50.18 ± 2.39 | 4.91 ± 2.33 |
| | **72** | 17.38 ± 4.00 | 28.51 ± 4.31 | 6.15 ± 0.70 | 34.06 ± 3.35 | 12.20 ± 1.85 |
| | **120** | 35.40 ± 5.84 | 22.11 ± 2.54 | 4.56 ± 1.19 | 22.94 ± 2.48 | 15.17 ± 3.74 |
| **C-2053** | **24** | 3.06 ± 1.09 | 22.34 ± 2.79 | 19.19 ± 1.77 | 44.81 ± 7.10 | 8.89 ± 1.38 |
| | **72** | 10.45 ± 2.56 | 35.18 ± 2.63 | 5.43 ± 1.59 | 36.32 ± 3.14 | 11.02 ± 2.20 |
| | **120** | 23.64 ± 4.86 | 30.06 ± 4.35 | 4.10 ± 0.56 | 27.20 ± 4.16 | 14.62 ± 4.62 |

**Table S2.** Cell cycle distribution in UAs treated H460 cells Flow cytometry analysis of cell cycle distribution of H460 untreated (control) and treated cells with C-2028, C-2041, C-2045 and C-2053 at concentrations corresponding to $IC_{90}$ doses for the times indicated. Data shows percentages of cells in the sub-G1, G1, S, G2/M, and Poli (polyploid cells) phases of the cell cycle. Data represents the averages of at least three independent experiments with standard deviation (n ≥ 3).

| | | Phase of Cell Cycle | | | | |
|---|---|---|---|---|---|---|
| **Compound** | **Incubation Time [h]** | **Sub-G1** | **G1** | **S** | **G2/M** | **Poli** |
| **Control** | **24** | 2.39 ± 0.73 | 60.09 ± 1.73 | 16.46 ± 0.55 | 18.37 ± 1.08 | 2.66 ± 0.64 |
| **C-2028** | **24** | 11.09 ± 3.80 | 32.92 ± 3.77 | 32.74 ± 2.09 | 21.64 ± 4.23 | 1.70 ± 0.42 |
| | **72** | 25.45 ± 7.20 | 37.15 ± 4.13 | 15.80 ± 2.33 | 19.21 ± 6.60 | 2.11 ± 0.55 |
| | **120** | 26.84 ± 1.92 | 29.87 ± 2.31 | 9.93 ± 2.46 | 28.98 ± 3.64 | 4.03 ± 0.95 |
| **C-2041** | **24** | 8.43 ± 3.14 | 31.30 ± 2.48 | 34.76 ± 4.78 | 23.89 ± 5.34 | 1.78 ± 0.42 |
| | **72** | 17.43 ± 2.40 | 42.86 ± 1.87 | 16.09 ± 2.94 | 21.24 ± 2.69 | 2.52 ± 0.71 |
| | **120** | 36.47 ± 3.18 | 35.08 ± 3.65 | 11.59 ± 1.16 | 14.84 ± 4.06 | 2.30 ± 0.60 |
| **C-2045** | **24** | 9.84 ± 4.46 | 32.25 ± 3.01 | 26.56 ± 3.44 | 28.15 ± 2.14 | 1.78 ± 0.33 |
| | **72** | 18.66 ± 3.61 | 39.94 ± 4.65 | 18.53 ± 5.99 | 20.87 ± 5.18 | 2.05 ± 0.60 |
| | **120** | 42.42 ± 8.29 | 23.89 ± 3.28 | 9.03 ± 1.37 | 21.49 ± 5.96 | 2.28 ± 1.33 |
| **C-2053** | **24** | 6.28 ± 1.52 | 30.44 ± 2.27 | 33.77 ± 3.66 | 27.37 ± 2.20 | 2.76 ± 0.30 |
| | **72** | 22.37 ± 2.06 | 36.08 ± 5.84 | 14.09 ± 2.46 | 24.96 ± 3.05 | 1.73 ± 0.52 |
| | **120** | 41.51 ± 5.77 | 25.01 ± 3.97 | 10.78 ± 1.76 | 19.77 ± 6.06 | 2.39 ± 1.17 |
